# Biochemical Activity of Vaborbactam

**DOI:** 10.1128/AAC.01935-19

**Published:** 2020-01-27

**Authors:** Ruslan Tsivkovski, Olga Lomovskaya

**Affiliations:** a Qpex Biopharma, Inc., San Diego, California, USA

**Keywords:** vaborbactam, beta-lactamase inhibitors, beta-lactamase, kinetics of inhibition

## Abstract

The most common mechanism of resistance to β-lactams antibiotics in Gram-negative bacteria is production of β-lactamase enzymes capable of cleaving the β-lactam ring. Inhibition of β-lactamase activity with small-molecule drugs is a proven strategy to restore the potency of many β-lactam antibiotics. Vaborbactam (formerly RPX7009) is a cyclic boronic acid β-lactamase inhibitor (BLI) with a broad spectrum of activity against various serine β-lactamases, including KPC carbapenemases. The combination of vaborbactam and meropenem is approved in the United States and Europe for the treatment of various nosocomial infections. We attempted to gain more insight into the mechanism of action of vaborbactam by conducting detailed kinetic characterization of its interaction with various recombinant His-tagged β-lactamases. Vaborbactam demonstrated potent inhibition of class A and class C enzymes with *K_i_* values ranging from 0.022 to 0.18 μM, while inhibition of class D enzymes was rather poor, and no activity against class B β-lactamases was detected. Importantly, vaborbactam inhibited KPC-2, KPC-3, BKC-1, and SME-2 carbapenemases at 1:1 stoichiometry, while these numbers were higher for other class A and C enzymes. Vaborbactam was also shown to be a potent progressive inactivator of several enzymes, including KPCs with inactivation constants *k*_2_/*K* in the range of 3.4 × 10^3^ to 2.4 × 10^4^ M^−1^ s^−1^. Finally, experiments on the recovery of enzyme activity demonstrated the high stability of the vaborbactam-KPC complex, with 0.000040 s^−1^ *k*_off_ values and a corresponding residence time of 7 h, whereas the release of vaborbactam bound to other serine β-lactamases was substantially faster. The biochemical characteristics of vaborbactam described in this study may facilitate further chemical optimization efforts to develop boronic BLIs with improved affinity and broader spectrum of inhibition.

## Abstract (precis)

The most common mechanism of resistance to β-lactams antibiotics in Gram-negative bacteria is production of β-lactamase enzymes capable of cleaving the β-lactam ring. Inhibition of β-lactamase activity with small-molecule drugs is a proven strategy to restore the potency of many β-lactam antibiotics.

## INTRODUCTION

The most common mechanism of resistance to β-lactams antibiotics in Gram-negative bacteria is production of β-lactamase enzymes capable of cleaving the β-lactam ring, resulting in complete loss of antibacterial activity. This family of enzymes has demonstrated tremendous growth over the past 2 decades and currently is represented by several structural classes (1). Of greatest concern is the recently observed spread of carbapenemase enzymes that can hydrolyze carbapenem antibiotics and threatens their clinical usefulness. In clinical settings, carbapenem-resistant *Enterobacteriaceae* (CRE) infections are associated with high rates of morbidity and mortality worldwide due to limited treatment options (2).

**FIG 1:**
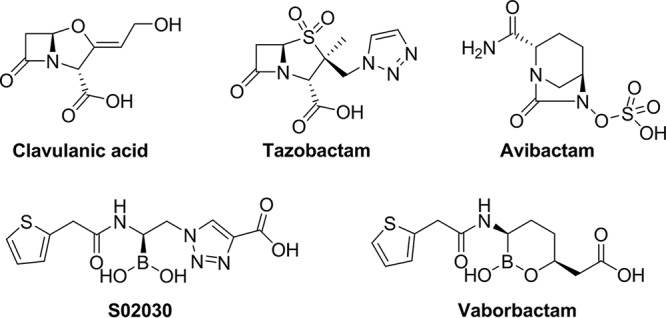
Chemical structures of various BLIs.

Inhibition of β-lactamase activity with small-molecule drugs is a proven strategy to restore the potency of many β-lactam antibiotics (3). The long-ago discovered β-lactamase inhibitors (BLIs) clavulanic acid and tazobactam (Fig. 1) are potent against various class A and class C enzymes but lack activity against many clinically relevant carbapenemases. Additional medicinal chemistry efforts resulted in development of a new non-β-lactam-based BLI avibactam (Fig. 1) possessing activity against numerous serine enzymes, including KPC carbapenemases (4). It has been approved for clinical use in combination with ceftazidime to treat complicated urinary tract infections (cUTIs), hospital-acquired and ventilator-associated bacterial pneumonia (HABP and VABP, respectively), and complicated intra-abdominal infections in combination with metronidazole (5). Several compounds based on the same structural core are now at various stages of preclinical or clinical development (6–8). However, resistance development to avibactam both *in vitro* and in clinical settings due to target *bla*_KPC_ mutations has been reported in multiple instances (9–12).

As an alternative structural scaffold, boronic acids have undergone extensive evaluation as inhibitors of serine β-lactamases due to the formation of a stable covalent bond between the boron moiety and the active-site serine residue (13–15). For instance, boronic acid BLI S02030 (Fig. 1) was demonstrated to target a wide variety of serine enzymes, including ADC-7, KPC-2, and SHV-1, with nanomolar potency (16, 17). Recently, our efforts to develop more efficient inhibitors of carbapenemases led to the discovery of vaborbactam (formerly RPX7009, Fig. 1), a cyclic boronic acid BLI with a broad spectrum of activity against class A and C β-lactamases (18). The most prominent feature of vaborbactam is its ability to inhibit KPC enzymes and to potentiate the activity of various carbapenems in CRE strains carrying these β-lactamases (19, 20). The combination of vaborbactam and meropenem is approved in the United States for the treatment of cUTI (21) and in Europe for the treatment of cUTI, complicated intra-abdominal infection, and HABP/VABP (22). Importantly, *in vitro* multistep resistance development studies with the meropenem-vaborbactam combination using numerous clinical isolates that harbored KPC failed to generate any target mutations in *bla*_KPC_ genes (23). In addition, there were no reports of resistance development due to mutations in β-lactamase genes after extensive use of the meropenem-vaborbactam combination in clinical settings.

The vaborbactam structure represents a promising chemical scaffold for further development of BLIs with improved properties and broader spectrum of activity, which requires a deep understanding of the structural and kinetic aspects of interaction of the BLI with its enzymatic targets. Crystal structures of vaborbactam with CTX-M-15 and AmpC β-lactamases revealed the spatial orientation of the inhibitor molecule in the active site, as well as a set of amino acid residues involved in interaction with the BLI (18). However, a detailed kinetic characterization of vaborbactam interaction with various β-lactamases has been missing so far. In this investigation we attempted to gain more insight into the mechanism of vaborbactam binding kinetics with various serine β-lactamases with a specific emphasis on carbapenemases.

## RESULTS AND DISCUSSION

Apparent *K_i_* (*K_i_*_app_) values of vaborbactam inhibition of various recombinant His-tagged β-lactamases from classes A, B, C, and D were measured using a procedure previously employed for boronic BLIs (13, 24) (Table 1). Vaborbactam demonstrated the ability to inhibit the majority of class A serine carbapenemases. The activity of KPC-2 and KPC-3 enzymes was inhibited with 0.056 ± 0.015 μM and 0.050 ± 0.016 μM *K_i_*_app_ values, respectively. Also, recently discovered BKC-1 and FRI-1 carbapenemases (25, 26) that share very low homology with other serine β-lactamases were inhibited by vaborbactam with 0.018 ± 0.002 μM and 0.17 ± 0.06 μM *K_i_*_app_ values, respectively. The *K_i_*_app_ value of inhibition of the SME-2 enzyme from S. marcescens was 0.042 ± 0.005 μM. In addition to being a potent inhibitor of class A cabapenemases, vaborbactam demonstrated inhibitory activity against several ESBL as well as AmpC enzymes with *K_i_* values varying from 0.021 to 1.04 μM (Table 1). Vaborbactam demonstrated relatively poor inhibition of class D β-lactamases OXA-48 and OXA-23 resulting with *K_i_*_app_ values of 14 ± 5 μM and 66 ± 11 μM, respectively. Similar low affinity to OXA enzymes was reported for various arylboronic acid derivatives (27, 28). The crystal structures of some of these BLIs with OXA-24/40 revealed covalent bond formation between the boron and catalytic serine residues, while various side chains attached to the arylboronate moiety were not involved in any specific interactions with the generally hydrophobic catalytic site of the enzyme (28). Perhaps the lack of such interaction in the vaborbactam-OXA complex could explain its significantly lower affinity. Interestingly, inhibition experiments with a series of arylcycloboronate BLIs revealed that one of them containing a cyclohexyl side chain inhibited various OXA enzymes with 50% inhibitory concentrations (IC_50_s) of 0.22 μM and lower (29), suggesting that the constrained cycloboronate scaffold may be more suitable for binding in the active site of class D enzymes.

**TABLE 1:** *K_i_*_app_ values of vaborbactam inhibition of various β-lactamases

Enzyme	Class	Carbapenemase	*K_i_*_app_ (μM)
KPC-2	A	**+**	0.056 ± 0.015
KPC-3	A	**+**	0.050 ± 0.016
BKC-1	A	**+**	0.018 ± 0.002
FRI-1	A	**+**	0.17 ± 0.06
SME-2	A	**+**	0.042 ± 0.005
CTX-M-14	A	**–**	0.033 ± 0.013
CTX-M-15	A	**–**	0.030 ± 0.004
SHV-12	A	**–**	0.021 ± 0.004
TEM-10	A	**–**	0.14 ± 0.04
TEM-43	A	**–**	1.04 ± 0.20
AmpC	C	**–**	0.035 ± 0.015
OXA-48	D	**+**	14 ± 5
OXA-23	D	**+**	66 ± 11
NDM-1	B	**+**	>160
VIM-1	B	**+**	>160

No inhibition of the class B metalloenzymes NDM-1 and VIM-1 by vaborbactam was detected. This result is not surprising given the absence of a serine residue in the active site to form a covalent bond with the inhibitor. However, several cycloboronate compounds were reported to inhibit NDM-1, VIM-2, and BclII enzymes with IC_50_ values ranging from 0.002 to 1 μM (29, 30), suggesting a different mode of binding in the active site. Cocrystallization studies indeed demonstrated interaction of the boron-bound oxygen atoms with the Zn1 ion in the substrate binding pocket, while the bicyclic benzoxaborinine ring creates hydrophobic interactions with the conserved Trp and Phe residues (30).

Mechanism-based suicidal BLIs (clavulanic acid, tazobactam, and sulbactam) function by acylating the catalytic serine residue of the enzyme (31). The resulting covalent complex can be hydrolyzed by a water molecule, similar to typical β-lactam substrates, that leads to release of an intact β-lactamase and the open ring form of the BLI molecule; alternatively, this complex can undergo structural rearrangements resulting in irreversible enzyme inactivation (32). The number of BLI molecules required to inactivate one molecule of β-lactamase is known as the stoichiometry of inactivation or partition ratio. Consequently, we determined the stoichiometry of inhibition of various serine enzymes that demonstrated a reasonable level of inhibition by vaborbactam (Table 2). KPC-2, KPC-3, BKC-1, and SME-2 were inhibited by vaborbactam at a 1:1 ratio, while CTX-M-15, FRI-1, and AmpC demonstrated 2:1, 8:1, and 16:1 stoichiometries of inhibition by vaborbactam, respectively. For SHV-12 and TEM-43 enzymes it was impossible to reach complete inhibition even at the highest (256:1) molar ratio. The >1 stoichiometry ratios are unlikely due to vaborbactam hydrolysis but rather because of the specific conditions of the experiment that require enzyme-BLI complex formation at 1 μM concentration of enzyme. Subsequent dilution of the reaction mixture to determine residual enzyme activity results in quick inhibitor dissociation due to high vaborbactam *k*_off_ rates for those enzymes (see below). Importantly, unlike many suicidal BLIs, no degradation of vaborbactam was observed after the inhibitor was incubated with KPC-2 for 18 h and subjected to subsequent liquid chromatography-mass spectrometry analysis (Fig. S1 in the supplemental material).

**TABLE 2:** Stoichiometry of vaborbactam inhibition of various β-lactamase enzymes

Enzyme	Stoichiometry^*a*^
KPC-2	1
KPC-3	1
BKC-1	1
FRI-1	8
SME-2	1
CTX-M-15	2
SHV-12	>256
TEM-43	>256
AmpC	16

*^a^*Stoichiometry, number of inhibitor molecules required to reduce enzyme activity by <10%.

**FIG 2:**
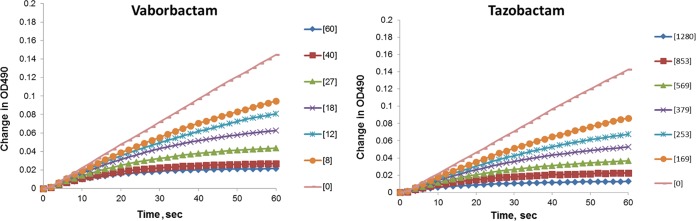
Kinetic profiles of KPC-2 inactivation by vaborbactam and tazobactam. Vaborbactam and tazobactam at the indicated concentrations (in μM) were quickly mixed with 1.2 nM KPC-2 enzyme and 100 μM NCF as the reporter substrate, and the absorbance at 490 nm was recorded immediately every 2 s using a plate reader.

Mechanism-based BLIs are characterized by a two-step kinetic reaction pathway of the inhibitor binding to the enzyme (31). When studied using the reporter substrate method, this is manifested by progressive enzyme activity inactivation shown in Fig. 2 for tazobactam with KPC-2 (right panel). In contrast, early reported boronic BLIs (e.g., *m*-tolylboronic acid and 2-formylphenylboronic acid) (33) showed a linear KPC-2 inactivation profile, indicating that equilibrium between enzyme and inhibitor is very quickly established (Fig. S2). However, boronic acid inhibitor S02030 (Fig. 1) that is structurally very similar to vaborbactam demonstrated kinetic behavior with KPC-2 and SHV-1 enzymes similar to tazobactam (17). This led us to hypothesize that vaborbactam may also exhibit progressive inactivation profiles typical of covalent irreversible or slow tight binding reversible inhibitors. Kinetics of KPC-2 inactivation by vaborbactam demonstrated a slow onset of inhibition and nonlinear reaction profiles (Fig. 2). Similar inactivation profiles were obtained for all of the other enzymes presented in Table 3. These results suggest that vaborbactam interaction with these β-lactamases follows a two-step kinetic mechanism. The first step is the formation of a noncovalent complex EI characterized by binding constant K. The second step is a covalent interaction between the catalytic Ser residue of the enzyme and the boron atom of vaborbactam to form the EI* complex. This second step is characterized by the first-order rate constant *k*_2_. Independent determination of these values was impossible due to the linear relationship between *k*_obs_ and vaborbactam concentration values up to the highest inhibitor concentration tested (Fig. S3). The inability to separately determine *K* and *k*_2_ values has been reported previously for various BLIs and β-lactamases from different structural classes (17, 34, 35). Therefore, the second-order rate constant *k*_2_/*K* for the onset of inhibition was calculated. Vaborbactam demonstrated comparable *k*_2_/*K* values of (5.5 ± 0.5) × 10^3^ and (6.7 ± 0.3) × 10^3^ M^−1^ s^−1^ of inactivation of the KPC-2 and KPC-3 enzymes, respectively (Table 3). FRI-1 and SME-2 were inactivated by vaborbactam with similar efficiency, while BKC-1, CTX-M-15, and AmpC demonstrated higher efficiencies of inactivation by vaborbactam with *k*_2_/*K* ranging from 1.2 × 10^4^ to 2.4 × 10^4^ M^−1^ s^−1^. Overall, the *k*_2_/*K* inactivation constants demonstrated only a 4-fold difference between the lowest and highest values and were consistent with the results reported for other boronic BLIs (17). Interestingly, vaborbactam showed linear inactivation profiles with the SHV-12 and TEM-43 enzymes, which is characteristic of “fast on–fast off” boronic BLIs (Fig. S2). This precluded calculation of the corresponding *k*_2_/*K* values (data not shown). It is quite likely that interaction of vaborbactam with these enzymes proceeded through simple one-step formation of a covalent complex between the catalytic serine residue and the boron atom of vaborbactam, which can be rapidly hydrolyzed by a water molecule to release intact vaborbactam.

**TABLE 3:** Kinetic parameters of vaborbactam inactivation of various β-lactamases

Enzyme	Mean ± SD			*K_d_* (nM)
	*k*_2_/*K* (M^−1^ s^−1^)	*k*_off_ (s^−1^)	Residence time (min)	
KPC-2	(5.5 ± 0.5) × 10^3^	0.000043 ± 0.000006	394 ± 50	7.8
KPC-3	(6.7 ± 0.3) × 10^3^	0.000030 ± 0.000001	559 ± 28	4.4
BKC-1	(1.2 ± 0.1) × 10^4^	0.00040 ± 0.00008	43 ± 8	33
FRI-1	(3.4 ± 0.1) × 10^3^	0.0017 ± 0.0001	9.8 ± 0.7	509
SME-2	(5.0 ± 0.2) × 10^3^	0.00024 ± 0.00002	71 ± 7	47
CTX-M-15	(2.3 ± 0.2) × 10^4^	0.0009 ± 0.0002	19 ± 1	40
AmpC	(2.4 ± 0.2) × 10^4^	0.0052 ± 0.0003	3.2 ± 0.2	220

**FIG 3:**
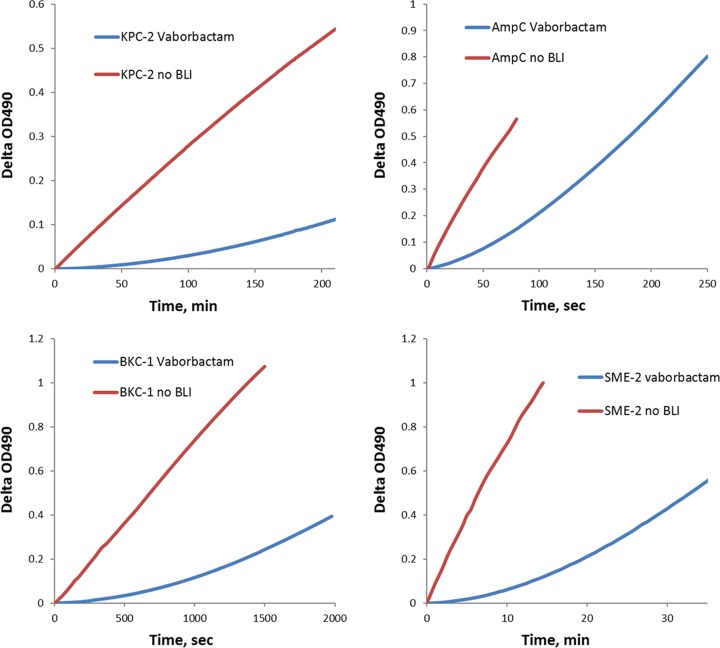
Kinetic profiles of activity recovery of various β-lactamases after inhibition by vaborbactam determined using the jump dilution method. Enzymes at 1 μM were mixed with vaborbactam at a concentration 8-fold higher than the stoichiometry ratio and then incubated for 30 min. After appropriate dilution, 100 μM NCF was added to the reaction mixture, and absorbance at 490 nm was recorded every 10 s using a plate reader. The reaction without the the addition of BLI was also recorded and used to calculate uninhibited enzyme velocity *V*_s_.

There have been no reports on the ability of boronic BLIs to induce structural rearrangements in β-lactamases that would result in irreversible enzyme modification as was reported for various β-lactam-like BLIs (31). In general, the chemical bond between the boron atom of the BLI and serine residues can be hydrolyzed by a water molecule, releasing intact inhibitor and enzyme. Taken together, this suggests that inhibition of β-lactamase enzymes by vaborbactam could be completely reversible upon removal of the BLI. The recovery of enzyme activity after complete inhibition by vaborbactam was studied by the jump dilution method (35). Activity recovery profiles for some enzymes are presented in Fig. 3. Unlike *k*_2_/*K* the inactivation constant, the calculated *k*_off_ values demonstrated a much higher degree of variation from 0.000030 s^−1^ for KPC-3 to 0.0052 s^−1^ for AmpC (Table 3). When these numbers were converted to residence time of enzyme-BLI complex, it resulted in a value of 559 ± 28 min for KPC-3 versus 3.2 ± 0.2 min for AmpC. Thus, vaborbactam forms remarkably stable complexes with KPC-2 and KPC-3 enzymes, while the stability of its complex with AmpC is substantially weaker. The *k*_off_ values for the SHV-12 and TEM-43 enzymes could not be determined by the jump dilution method due to inability to completely inhibit their activity by vaborbactam, even at a very high 256:1 molar ratio. Next, *K_d_* values were calculated using *k*_2_/*K* and *k*_off_ kinetic parameters (Table 3). They ranged from 4.4 nM for the KPC-3 enzyme and up to 509 nM for FRI-1. Such a high degree of variation can be attributed to the wide range of *k*_off_ values, whereas the difference between *k*_2_/*K* values was not as dramatic. Interestingly, for KPC-2 and KPC-3 enzymes the *K_d_* values were almost 10-fold lower than the corresponding *K_i_*_app_ values (Table 1). The difference could be attributed to the fact that *K_i_*_app_ measurements were done after enzyme incubation with BLI for 10 min, while *K_d_* values reflect enzyme affinity at equilibrium.

In summary, we demonstrated that vaborbactam is a potent inhibitor of various serine β-lactamases belonging to structural classes A and C, including several carbapenemases. Of particular importance is the inhibition of clinically relevant KPC carbapenemases, which contribute very strongly to the worldwide spread of CRE infections (36). In addition, three structurally diverse carbapenemase enzymes—SME-2, BKC-1, and FRI-1—are also inhibited by vaborbactam with reasonable potency. Regarding the mechanism of inhibition, with the majority of the tested enzymes, vaborbactam behaves as a slow tight-binding inhibitor that requires two distinct kinetic steps for enzyme inactivation. Crystal structures of vaborbactam complexes with CTX-M-15 and AmpC β-lactamases demonstrated an extensive interaction network formed between the amino acid residues surrounding the enzyme substrate binding pocket and the carboxy and amide groups of the inhibitor molecule (18). One might speculate that this interaction could possibly contribute to the biphasic kinetic behavior of vaborbactam with certain enzymes. Another important finding in this study is the almost irreversible binding of vaborbactam to the KPC carbapenemases, with calculated residence times reaching several hours. Comparison of molecular structures of vaborbactam complexes with KPC-2 (unpublished data) versus other enzymes may shed light on the structural elements responsible for this phenomenon. The increased residence time of the BLI-enzyme complex may also have a positive effect on the *in vivo* potency of vaborbactam in animal infection models (37). Overall, the biochemical characteristics of vaborbactam described in this study will be useful for further chemical optimization efforts to develop boronic BLIs with improved affinity and a broader spectrum of inhibition.

## MATERIALS AND METHODS

### Purification of KPC-2 and KPC-3 proteins for biochemical studies.

Full KPC-2 and KPC-3 gene coding sequences were cloned into pET28a vector that produced an expression construct with periplasmic protein secretion and 6×His tag on its C terminus. The recombinant plasmids were transformed into BL21(DE3)pLys strain. First, 2 ml of overnight culture was inoculated in 1 liter of Luria-Bertani (LB) medium with 50 μg/ml of kanamycin and 20 μg/ml of chloramphenicol and grown at 37°C with 300-rpm shaking until reaching an optical density at 600 nm (OD_600_) of 0.7 to 0.8. IPTG (isopropyl-β-d-thiogalactopyranoside) was added to 0.2 mM, and the cells continued to grow for an additional 3 h. The cells were harvested by centrifugation, and the pellet was resuspended in 40 ml of ice-cold 50 mM Tris-HCl (pH 8.0), 500 mM sucrose, 1 mM EDTA, and one tablet of complete protease inhibitor (Roche-Sigma-Aldrich, St. Louis, MO). The suspension was incubated on ice with six cycles of 15-s vortexing with a 5-min pause between cycles. The suspension was centrifuged for 30 min at 30,000 × *g*. The supernatant was collected and sonicated for 30 s to reduce the viscosity, and MgCl_2_ and imidazole were added to 2 and 5 mM, respectively. The lysate was loaded by gravity flow onto a 1-ml column with HisPur cobalt resin (Thermo Scientific) preequilibrated with 50 mM sodium phosphate (pH 7.4)–300 mM NaCl–5 mM imidazole buffer. The column was washed with 40 ml of the same buffer, and then His tag protein was eluted with 50 mM sodium phosphate (pH 7.4)–300 mM NaCl–70 mM imidazole buffer. All wash and elution fractions were analyzed by 8 to 16% SDS-PAGE. Fractions containing target protein were pooled, concentrated, and dialyzed against 50 mM sodium phosphate (pH 7.0). The purity of all proteins was at least 95% as determined by SDS-PAGE. Protein preparations were aliquoted and stored at –20°C until further use.

### Purification of OXA-23, BKC-1, FRI-1, and SME-2 proteins for biochemical studies.

The coding sequences for all four proteins were cloned into a pET28a vector that produced an expression construct with cytoplasmic protein localization and an N-terminal 6×His tag. The recombinant plasmids were transformed into the BL21(DE3) strain. Then, 25 ml of overnight culture grown in LB medium at 30°C was inoculated into 1 liter of MagicMedia (Thermo Fisher Scientific) with 25 μg/ml of kanamycin and grown at 18°C with 300-rpm shaking for 32 h. The cells were harvested by centrifugation, and the pellet was resuspended in 40 ml of ice-cold 50 mM sodium phosphate (pH 7.5), 300 mM NaCl, and one complete protease inhibitor tablet. The suspension was subjected to six cycles of 1-min sonication with a 5-min pause between each cycle on ice. The suspension was centrifuged for 1 h at 30,000 × *g*, the supernatant was collected, and imidazole was added to 5 mM. Further enzyme purification was performed using the same approach as that used with KPC-2 and KPC-3.

All other enzymes used in the study were expressed and purified by Emerald Biostructures (Bainbridge Island, WA).

### Determination of vaborbactam *K_i_* values for various β-lactamases.

The protein was mixed with various concentrations of inhibitors in 50 mM sodium phosphate (pH 7.0) and 0.1 mg/ml of bovine serum albumin buffer (reaction buffer), followed by incubation for 10 min at 37°C. Next, 50 μM nitrocefin (NCF; 10 μM for SHV-12 and 25 μM for BKC-1) was added, and substrate cleavage profiles were recorded at 490 nm every 10 s for 10 min. NCF concentrations for *K_i_* determinations were selected not to exceed *K_m_* values by >2-fold to prevent the “saturation” of enzyme activity with substrate. *K_i_*_app_ values were calculated by method of Waley (24).

### Stoichiometry of β-lactamase inhibition by vaborbactam.

Enzyme at 1 μM in reaction buffer was mixed with BLI at molar ratios varying from 256 to 0.0625. After 30 min of incubation at 37°C, the reaction mixture was diluted 200-fold, and the enzyme activity was measured with NCF as described above. Stoichiometry of inhibition was determined as the minimal BLI/enzyme ratio reducing enzyme activity to <10%.

### Determination of the vaborbactam *k*_2_/*K* inactivation constant for various β-lactamases.

Inactivation kinetic parameters were determined by using the reporter substrate method for the slow tight-binding inhibitor kinetic scheme (38).

$$\begin{array}{c} \text{E}+\text{I}\overset{K}{↔}\;\text{EI}\overset{k_{2}}{\underset{k_{-2}}{↔}}\text{ EI}* \end{array}$$

Protein was quickly mixed with 100 μM nitrocefin and various concentrations of BLI in reaction buffer, and the absorbance at 490 nm was measured immediately every 2 s for 180 s on a SpectraMax plate reader (Molecular Devices, San Jose, CA) at 37°C. The resulting progression curves of OD_490_ versus time at various BLI concentrations were imported into Prism software (GraphPad, San Diego, CA), and pseudo first-order rate constants *k*_obs_ were calculated by using the following equation: *P* = *V_s_* ⋅ (1 – e^–^*^k^*^obs ⋅^ *^t^*)/*k*_obs_, where *V_s_* is the enzyme NCF cleavage rate in the absence of BLI. The *k*_obs_ values calculated at various vaborbactam concentrations were fitted in the following equation:

$$k_{\text{obs}}=k_{-\text{2}}+k_{\text{2}}/K\times \left[\text{I}\right]/\left(\text{1}+\left[\text{NCF}\right]/K_{m}\left[\text{NCF}\right]\right)$$

where *k*_2_/*K* is the inactivation constant, [I] is the inhibitor concentration, and [NCF] is the nitrocefin concentration.

### *K_m_* (NCF) Michaelis constant of NCF for β-lactamase.

The following enzyme NCF *K_m_* values were used for *k*_2_/*K* calculation: KPC-2, 49 ± 5 μM; KPC-3, 49 ± 2 μM; BKC-1, 9.4 ± 1.4 μM; FRI-1, 75 ± 4 μM; SME-2, 59 ± 3 μM; CTX-M-15, 24 ± 3 μM; and AmpC, 33 ± 3 μM.

### Determination of *k*_off_ rates of enzyme activity recovery after inhibition by vaborbactam.

Purified enzyme at 1 μM in reaction buffer was mixed with BLIs at an 8-fold higher concentration than its stoichiometry ratio (determined in preliminary stoichiometry experiments). After 30 min incubation at 37°C, the reaction mixture was diluted from 100- to 30,000-fold depending on the enzyme in reaction buffer, and 100 μl of diluted enzyme was mixed with 100 μl of 400 μM NCF in reaction buffer. The absorbance at 490 nm was recorded every minute during 4 h at 37°C. The resulting reaction profiles were fitted into the following equation using Graph Pad Prism software to obtain *k*_off_ values: *P* = *V*_s_ × *t* + (*V*_o_ – *V*_s_) × (1 – e^–^*^k^*^off ⋅ t^)/*k*_off_, where *V*_s_ is the uninhibited enzyme velocity, measured in the reaction with enzyme and no inhibitor, and *V*_o_ is the completely inhibited enzyme velocity, measured in the reaction with no enzyme and NCF only.

### Statistical analysis.

All kinetic results are presented as averages ± the standard deviations of minimum three replicates.

## Supplementary Material

Supplemental file 1
