# Supplementary material for: Biochemical Activity of Vaborbactam
Source: Antimicrob Agents Chemother. 2020 Jan 27;64(2):e01935-19. doi: 10.1128/AAC.01935-19 (PMC6985712; doi:10.1128/AAC.01935-19)
Supplement: Supplemental file 1 [file AAC.01935-19-s0001.pdf]

Supplemental Material FOR Publication

Supplementary figures

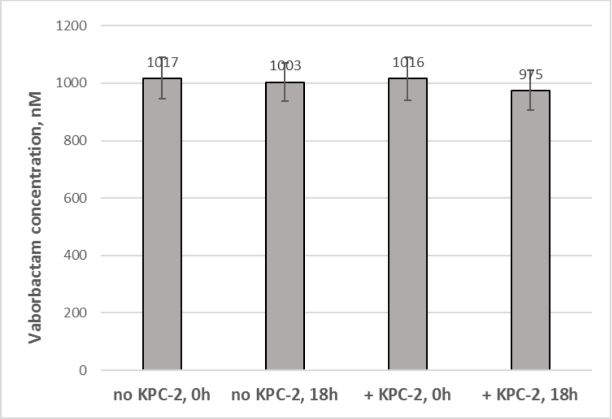

Figure S1. Stability of vaborbactam after incubation with KPC-2. 1 μM of vaborbactam and 1 μM KPC-2 were incubated at 37ºC in reaction buffer for 18 hours. Aliquotes of reaction mixture were taken before and after the incubation and analyzed by LC/MS. Vaborbactam incubated for 18 hours without KPC-2 was used as negative control. Vaborbactam was not hydrolyzed by KPC-2. At the same conditions tazobactam was completely degraded in 1 hour (data not shown). Kinetic analysis demonstrated that KPC-2 retained its full activity after 18 hours of incubation in the reaction buffer (data not shown).

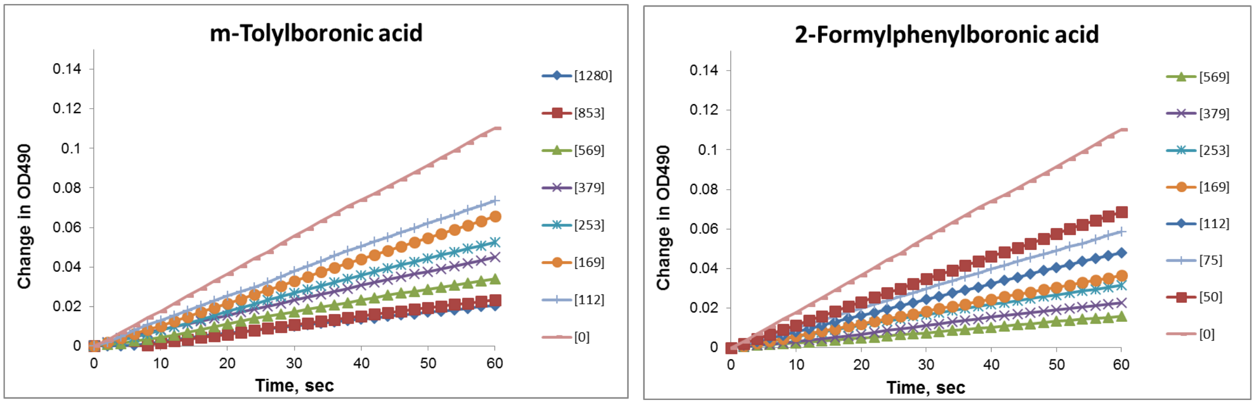

Figure S2. Kinetic profiles of KPC-2 inactivation by m-tolylboronic acid and 2-formylphenylboronic acid. Compounds at indicated concentration (in μM) were quickly mixed with 1.2 nM KPC-2 enzyme and 100 μM NCF as reported substrate and absorbance at 490 nm was recorded immediately every 2 sec using plate reader.

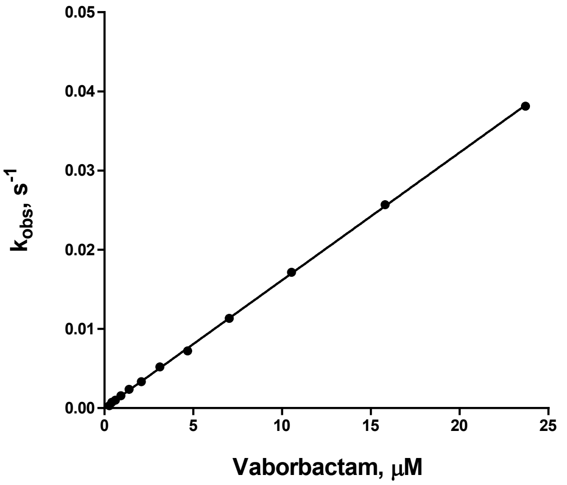

Figure S3. Plot of k_{obs} vs vaborbactan concentration after KPC-2 inactivation studied by reporter substrate method. Vaborbactam at indicated concentration (in μM) was quickly mixed with 1.2 nM KPC-2 enzyme and 100 μM NCF as reported substrate and absorbance at 490 nm was recorded immediately every 2 sec using plate reader. Resulting absorbance profiles were used to calculate k_{obs} values as **described** in Materials and Methods.

Supplementary Materials and Methods

Determination of vaborbactam concentration in KPC-2 treated samples by liquid chromatography with tandem mass spectrometry (LC-MS/MS). Vaborbactam was dissolved in 50-50 water-acetonitrile at a concentration of 5.00 mg/mL and diluted in buffer A to prepare a calibration curve covering the range of concentrations from 40.0 to 800 ng/mL, together with appropriate reagent and matrix blanks. 50 μl of each calibration standard were aliquoted into a 1.5 mL vial and combined with 150 μl of 100% MeOH with 20 ng/ml of a standard. Samples,

standards and blanks were centrifuged and the supernatant was diluted in water, then stored at 5ºC pending injection on LC-MS.

Sample and standard extracts were analyzed using an Agilent 1100 HPLC binary pump and degasser with column compartment coupled to an AB Sciex 3200 QTrap with ESI source operated in the positive ion mode. Mobile phase A was 0.1% formic acid in water; mobile phase B was 0.1% formic acid in acetonitrile. Separation was achieved using a Waters XBridge Shield 2.1x50mm column with 5 µm particles operated in isocratic mode with 88% mobile phase A and 12% mobile phase B. The mass transitions used for the analyte and the internal standard were 279.991 > 220.2 and 260.046 > 200 respectively. The peak area ratio of analyte / IS was used for quantitation, and the calibration standards were fit to a linear regression model with $1/x^2$ weighting using AB Sciex Analyst version 1.6 software.
